# Profiling Early Lung Immune Responses in the Mouse Model of Tuberculosis

**DOI:** 10.1371/journal.pone.0016161

**Published:** 2011-01-13

**Authors:** Dongwan D. Kang, Yinyao Lin, Javier-Rangel Moreno, Troy D. Randall, Shabaana A. Khader

**Affiliations:** 1 Department of Biostatistics, University of Pittsburgh, Pittsburgh, Pennsylvania, United States of America; 2 Division of Infectious Diseases, Department of Pediatrics, University of Pittsburgh School of Medicine, Pittsburgh, Pennsylvania, United States of America; 3 Division of Allergy, Immunology and Rheumatology, Department of Medicine, University of Rochester Medical Center, Rochester, New York, United States of America; Louisiana State University, United States of America

## Abstract

Tuberculosis (TB) is caused by the intracellular bacteria *Mycobacterium tuberculosis*, and kills more than 1.5 million people every year worldwide. Immunity to TB is associated with the accumulation of IFNγ-producing T helper cell type 1 (Th1) in the lungs, activation of *M.tuberculosis*-infected macrophages and control of bacterial growth. However, very little is known regarding the early immune responses that mediate accumulation of activated Th1 cells in the *M.tuberculosis*-infected lungs. To define the induction of early immune mediators in the *M.tuberculosis*-infected lung, we performed mRNA profiling studies and characterized immune cells in *M.tuberculosis*-infected lungs at early stages of infection in the mouse model. Our data show that induction of mRNAs involved in the recognition of pathogens, expression of inflammatory cytokines, activation of APCs and generation of Th1 responses occurs between day 15 and day 21 post infection. The induction of these mRNAs coincides with cellular accumulation of Th1 cells and activation of myeloid cells in *M.tuberculosis*-infected lungs. Strikingly, we show the induction of mRNAs associated with Gr1^+^ cells, namely neutrophils and inflammatory monocytes, takes place on day 12 and coincides with cellular accumulation of Gr1^+^ cells in *M.tuberculosis*-infected lungs. Interestingly, *in vivo* depletion of Gr1^+^ neutrophils between days 10–15 results in decreased accumulation of Th1 cells on day 21 in *M.tuberculosis*-infected lungs without impacting overall protective outcomes. These data suggest that the recruitment of Gr1^+^ neutrophils is an early event that leads to production of chemokines that regulate the accumulation of Th1 cells in the *M.tuberculosis*-infected lungs.

## Introduction

Tuberculosis (TB), caused by the intracellular bacteria *Mycobacterium tuberculosis*, is a global disease that kills more than 1.5 million people every year worldwide. Approximately one-third of the world's population is infected with *M.tuberculosis* and about 5–10% of these individuals will develop clinical disease over their lifetime [1]. In addition, development of drug-resistant strains of *M.tuberculosis* and increased incidence of HIV-associated TB threatens to overwhelm the current measures for TB control [2]. The only vaccine available against TB is BCG, which has variable efficacy in different populations. Thus, there is an urgent need to develop more effective vaccines against TB [1]. However, a significant hurdle in design of new vaccines is the limited understanding of the requirements for early immune responses to TB in the lung.

Immunity to TB is dependent on the accumulation of IFNγ-producing T helper cell type 1 (Th1) in the lungs, subsequent activation of *M.tuberculosis*-infected macrophages and control of bacteria [3]. The cytokine IL-12, made up of IL-12p35 and IL-12p40 subunits is crucial for the induction of IFNγ in Th1 cells [4]. Accordingly, humans and mice with mutations in the IL-12/Th1 pathway are more susceptible to mycobacterial infections [5], [6], [7]. In the mouse model of tuberculosis, the adaptive T cell immune responses are detected in the lung between day 18 and day 21 [8], [9], [10] and coincide with control of bacteria [9]. However, very little is known regarding the immune events that occur prior to accumulation of Th1 cells in the *M.tuberculosis*-infected lungs.

To define the induction of early immune mediators in the *M.tuberculosis*-infected lung, we have carried out mRNA profiling and characterized immune cells in *M.tuberculosis*-infected lungs at early stages of infection. Our data show that induction of genes involved in pathogen recognition, expression of inflammatory cytokines and activation of APCs takes place between days (D) 15 and 21 in *M.tuberculosis*-infected lungs. Also, we show that induction of mRNAs related to the IL-12/Th1 and TNF-alpha (TNFα) pathways occurs between D15–D21 post infection and coincides with cellular accumulation of Th1 cells and activation of myeloid cells in *M.tuberculosis*-infected lungs. Strikingly, we show the induction of mRNAs associated with Gr1^+^ cells, namely neutrophils and inflammatory monocytes, takes place on D12, and precedes the induction of mRNAs associated with the IL-12/Th1 pathway. We further confirm that Gr1^+^ cells accumulate in *M.tuberculosis*-infected lungs on D12 and show that depletion of Gr1^+^ neutrophils between days 10–15 results in decreased accumulation of Th1 cells in the *M.tuberculosis*-infected lungs on D21. However, decreased Th1 responses did not impact activation of myeloid cells in *M.tuberculosis*-infected lungs or bacterial control. Together, our data suggest that the recruitment of Gr1^+^ cells occurs prior to the accumulation of Th1 cells in *M.tuberculosis*-infected lungs and may regulate Th1 immune responses, but does not change overall protective outcomes.

## Materials and Methods

### Animals

C57BL/6 (B6) mice were purchased from The Jackson Laboratory (Bar Harbor, ME). Experimental mice were age and sex matched and used between the ages of eight to ten weeks. All mice were used in accordance with University of Pittsburgh Institutional Animal Care and Use Committee guidelines and were approved under Protocol 0807913. All efforts were made to minimize suffering and pain as described in this approved protocol.

### Experimental infection and Gr1 depletion

The H37Rv strain of *M.tuberculosis* was grown in Proskauer Beck medium containing 0.05% Tween 80 to mid-log phase and frozen in 1 ml aliquots at −70°C. For aerosol infections, subject animals were infected with ∼100 bacteria using a Glas-Col (Terre Haute, IN) airborne infection system as described in detail [11]. For depletion of Gr1^+^ neutrophils, mice were treated with 300 µg of Gr1 depleting antibody (Clone IA8, BioXcell) or isotype control antibody (BioXcell) every 48 hours. Bacterial burden was estimated by plating the lung homogenates on 7H11 agar plates.

### RNA extraction and microarray analysis

RNA was extracted from *M.tuberculosis*-infected and control uninfected lungs as previously described [12]. RNA samples were reverse transcribed to generate cDNA. cDNAs from uninfected (Control) and *M.tuberculosis*-infected lungs from each time point were hybridized to microarrays 45000 mouse probes representing 21308 genes. Expression values were normalized with GeneChip Robust Multiarray Average (GCRMA) [13]. Both raw data and the expression data set have been submitted to the NCBI Gene Expression Omnibus repository (GEO) with accession number GSE23014. Rank Product [14] was used to detect differentially expressed genes and to calculate False Discovery Rate (FDR). Rank Product was reported to perform well in small sample sizes and highly noise data set [15]. All analyses were performed with R and Bioconductor [16]. Functional Analysis using Gene Ontology terms for biological processes was performed with DAVID (http://david.abcc.ncifcrf.gov). Ingenuity Systems Pathway Analysis (http://www.ingenuity.com) was used for pathway analysis.

#### Cell Preparation and flow cytometry

Lung cell suspensions were prepared as described before [12] and used for flow cytometric analyses. Briefly, a single cell suspension was prepared from either digested lung tissue by dispersing the tissue through a 70µm nylon tissue strainer (BD Falcon, Bedford, MA). The resultant suspension was treated with Gey's solution to remove any residual red blood cells, washed twice and counted. Single cell suspensions were then stained with fluorochrome-labeled antibodies specific for CD3 (17A2), CD4 (RM4-5), Gr1 (RB6-8C5), CD11b (M1/70), CD11c (HL3), and MHC class II I-A^b^ (AF6-120.1), CD44 (IM7). For intracellular cytokine detection, cells were stimulated with Phorbol myristate acetate (50 ng/ml), ionomycin (750 ng/ml; Sigma Aldrich) and Golgistop (BD Pharmingen), were surface stained, permeabilized with Cytofix-Cytoperm solution (BD Pharmingen) and stained with anti-IFNγ antibody (XMG1.2). Cells were collected on a Becton Dickinson LSRII flow cytometer using FACS Diva software. For analysis, FlowJo (Tree Star Inc, CA) was used for cell analysis. Differences between the means of experimental groups were analyzed using the two tailed Student's *t*-test. Differences were considered significant when *p*≤0.05.

### Morphometric analysis and immunofluorescence

The lower right lobe of each lung was inflated with 10% neutral buffered formalin and processed routinely for light microscopy by hematoxylin and eosin stain (Colorado Histoprep, Fort Collins, CO). For immunofluorescence, paraffin was removed from the formalin-fixed lung sections, which were then washed with xylene, alcohol and PBS. Antigens were unmasked using a DakoCytomation Target Retrieval Solution and were blocked with 5% (v/v) normal donkey serum and Fc block. Endogenous biotin was neutralized with avidin followed by biotin (Sigma Aldrich). Sections were probed with goat anti mouse CD3ε to detect CD3 lymphocytes (clone M-20; Santa Cruz Biotechnology), and biotinylated Gr1 to detect neutrophils (Rat, BD Pharmingen) in the inflammatory lesions. Primary antibodies were detected with secondary antibody conjugated to Alexa flour 568 for CD3 (Alexa fluor 568, Donkey anti goat; Invitrogen). Gr1^+^ cells were visualized by adding Alexa fluor-488 and Streptavidin-alexa fluor 488 (Invitrogen). Slow fade gold antifade with DAPI (Molecular probes, Eugene, OR) was used to counterstain tissues and to detect nuclei. Images were obtained with a Zeiss Axioplan 2 microscope and were recorded with a Zeiss AxioCam digital camera.

## Results

### Global changes of gene expression during the early stages of *M.tuberculosis* infection

**Figure 1:**
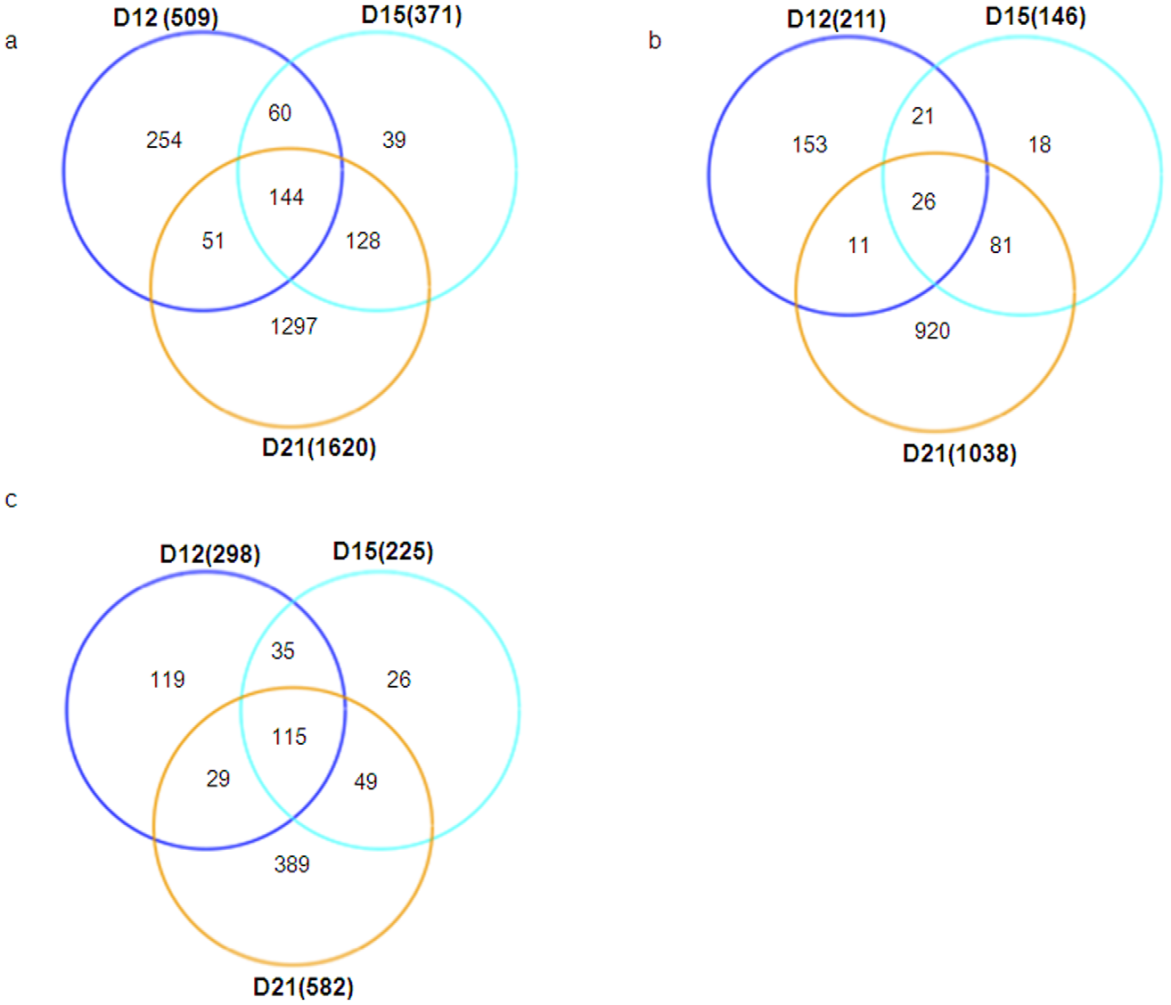
Global changes of gene expression during the early stages of *M.tuberculosis* infection. B6 mice were infected with ∼100 CFU *M.tuberculosis* via the aerosol route and at specific times after infection, lung tissue was harvested and processed to extract RNA. cDNA microarray hybridization was used to study gene expression profiles on infected lung cDNA at day 12 (D12 n = 4), day 15 (D15 n = 4) or day 21 (D21 n = 5) post infection. For each time point, cDNA was hybridized from uninfected (n = 3) and *M.tuberculosis*-infected lungs to microarrays containing 45000 mouse probes representing 21308 genes. Total genes differentially regulated at day 12 (D12) compared to day 15 (D15) and day 21 (D21) post infection was determined after controlling FDR at the level of 0.05 by using the R package “RankProd”. (a). Genes upregulated (b) or repressed (c) are also shown.

Gonzalez-Juarrero et al. have studied the gene expression of *M.tuberculosis*-infected lungs during the chronic phase of infection [17]. However, since accumulation of antigen-specific T cells and control of bacteria in the mouse model of tuberculosis takes place between days 15–20 post infection, we determined the global transcriptional responses in the lungs during the early immune response following *M.tuberculosis* infection. To address this, C57BL6 (B6) mice were aerosol-infected with ∼100 CFU of *M.tuberculosis* and cDNA microarray hybridization was used to study gene expression profiles on infected lung cDNA at different time points during the early immune response. For each time point, cDNA from uninfected and *M.tuberculosis*-infected lungs were hybridized to microarrays containing 45000 mouse probes representing 21308 genes. Upon comparison to mRNA from uninfected lungs, 509 mRNAs from *M.tuberculosis*-infected lungs were differentially expressed at day 12 (D12), 371 mRNAs on day 15 (D15) and 1620 mRNAs on day 21 (D21) post infection (Figure 1a). Not surprisingly, many of the same mRNAs were differentially expressed at multiple time points. For example, 204 mRNAs were similarly induced or repressed on D12 and D15, 272 mRNAs on D15 and D21 and 195 mRNAs on D21 and D12. Also, 144 mRNAs were similarly induced or repressed at all three time points. Approximately half of the differentially regulated transcripts were induced (Figure 1b), whereas half were repressed (Figure 1c) on D12 and D15. However, more genes were induced (Figure 1b) than repressed at D21 (Figure 1c).

**Figure 2:**
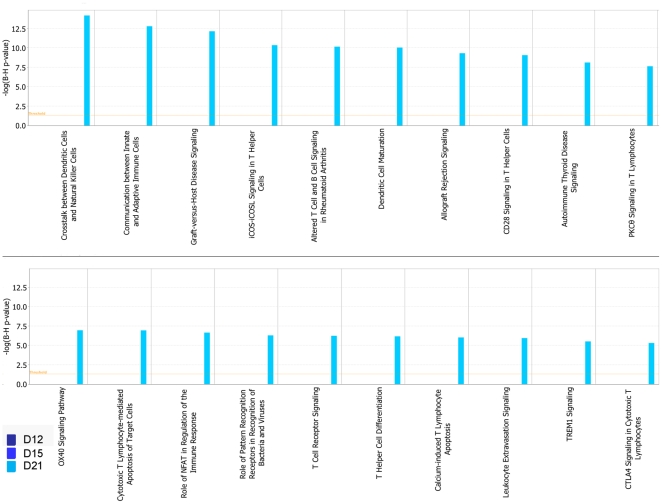
Induction of key immune pathways in mouse model of *M.tuberculosis* infection take places on D21 post infection. B6 mice were infected with ∼100 CFU *M.tuberculosis* via the aerosol route and at specific times after infection, lung tissue was harvested and processed to extract RNA. cDNA microarray hybridization was used to study gene expression profiles on uninfected lungs (n = 3), *M.tuberculosis*-infected lung cDNA at day 12 (D12 n = 4), day 15 (D15 n = 4) or day 21 (D21 n = 5) post infection and ingenuity signaling software was used to assess the induction of genes mainly induced on D21. Pathways induced are shown. The threshold represents 0.05 B–H adjusted p-value which was calculated from hypergeometric distribution. Longer bar represents a larger proportion of genes were enriched in a specific pathway.

We then determined whether transcripts in particular pathways were coordinately regulated using Ingenuity Signaling software. We found that most groups of transcripts associated with inflammation, dendritic cell maturation and T helper function showed significant alterations in expression only on D21 post infection (Figure 2). However, a group of mRNAs included those encoding the host heat shock proteins (Table 1) were induced at D12. Overall, these data suggest that there is a dynamic regulation of mRNA expression during the early stages of infection and that while many pathways are induced at later times after infection (D21), mRNAs belonging to the host heat shock proteins are induced early and likely contribute to stabilization of protein induced in response to infection-related stress.

**Table 1:** Induction of genes associated with heat shock proteins during early *M.tuberculosis* infection.

EntrezID	Symbol	GeneName	D12		D15		D21	
			FC	q-val	FC	q-val	FC	q-val
15511	HSPA1B	heat shock protein 1B	**6.36**	**0.00**	1.28	0.27	1.44	0.51
193740	HSPA1A	heat shock protein 1A	**3.80**	**0.00**	1.03	1.08	−1.02	0.67
14828	HSPA5	heat shock protein 5	1.24	0.30	**1.60**	**0.08**	**2.58**	**0.03**
15512	HSPA2	heat shock protein 2	**1.44**	**0.08**	−1.01	1.29	−1.15	1.24
50497	HSPA14	heat shock protein 14	**1.51**	**0.09**	1.21	0.52	1.55	0.20

### Regulated expression of mRNAs involved in innate immune responses

The engagement of pathogen-associated molecular pattern receptors is important for the initiation of innate immune responses. Toll like receptors (TLRs) are key components of the innate immune responses during mycobacterial infections, since induction of inflammatory cytokines following *M.tuberculosis* infection requires signals from TLR2 [18], [19], TLR4 [20] and cooperation between TLR2 and TLR9 [21]. Amongst the TLR genes, we found that mRNA transcripts for TLR 1, 2, 12 and 13, were significantly induced at day 21 (Table 2), while induction of TLR4 or TLR9 was not detected on D21 post infection. CD14, a TLR-associated protein required for macrophage activation following *M.tuberculosis* infection [22] was induced at D21, but not earlier in the infected lung. These data suggest that different TLRs may be functioning at different phases during the early stages of *M.tuberculosis* infection.

**Table 2:** Induction of genes associated with innate host immune mechanisms during early *M.tuberculosis* infection.

EntrezID	Symbol	GeneName	D12		D15		D21	
			FC	q-val	FC	q-val	FC	q-val
21897	TLR1	toll-like receptor 1	−1.51	0.33	−1.07	0.78	**6.94**	**0.00**
24088	TLR2	toll-like receptor 2	−1.18	0.94	1.01	1.36	**3.72**	**0.01**
21898	TLR4	toll-like receptor 4	−1.04	1.19	1.10	0.75	1.19	0.80
81897	TLR9	toll-like receptor 9	1.00	1.09	1.05	1.12	1.00	1.63
384059	TLR12	toll-like receptor 12	−1.01	1.39	1.00	1.09	**5.30**	**0.00**
279572	TLR13	toll-like receptor 13	−1.34	0.63	1.02	0.95	**4.61**	**0.00**
12475	CD14	CD14 antigen	1.17	0.43	1.04	1.09	**3.45**	**0.01**
170786	CD209A	CD209a antigen	1.29	0.20	−1.10	1.01	**−8.51**	**0.00**
100702	MPA2L	macrophage activation 2 like	−1.11	1.02	**1.73**	**0.04**	**18.36**	**0.00**
73149	CLEC4A3	C-type lectin domain family 4, member a3	−1.14	0.92	−1.02	1.15	**2.41**	**0.03**
56619	CLEC4E	C-type lectin domain family 4, member e	1.41	0.14	**2.69**	**0.00**	**236.47**	**0.00**
56620	CLEC4N	C-type lectin domain family 4, member n	−1.03	1.14	1.34	0.26	**3.97**	**0.01**
56644	CLEC7A	C-type lectin domain family 7, member a	1.04	0.97	**1.52**	**0.09**	**3.92**	**0.01**
23845	CLEC5A	C-type lectin domain family 5, member a	**1.56**	**0.05**	**1.94**	**0.03**	**18.62**	**0.00**
16409	ITGAM	integrin alpha M	1.17	0.55	1.04	0.72	**5.14**	**0.00**
16411	ITGAX	integrin alpha X	−1.30	0.68	−1.07	1.32	**2.37**	**0.03**
17386	MMP13	matrix metallopeptidase 13	1.02	1.03	1.01	1.22	**13.47**	**0.00**
17395	MMP9	matrix metallopeptidase 9	**1.51**	**0.06**	1.01	0.92	1.11	1.14
17394	MMP8	matrix metallopeptidase 8	1.44	0.12	1.05	0.94	1.85	0.15
17384	MMP10	matrix metallopeptidase 10	1.00	1.09	1.00	1.09	1.25	0.47
16189	IL4	interleukin 4	1.00	1.09	1.00	1.09	1.00	1.21
16193	IL6	interleukin 6	−1.00	1.39	1.45	0.22	**53.96**	**0.00**
16153	IL10	interleukin 10	1.00	1.09	1.00	1.09	1.08	1.09
16159	IL12A	interleukin 12a	1.15	0.46	1.07	0.74	−1.90	0.34
16160	IL12B	interleukin 12b	1.05	1.06	1.41	0.26	**14.54**	**0.00**
16161	IL12RB1	interleukin 12 receptor, beta 1	−1.00	1.38	1.02	1.25	**20.04**	**0.00**

We also observed the altered expression of other types of pathogen recognition receptors after *M.tuberculosis* infection. Interestingly, we found that the mRNA encoding DC-SIGN (CD209A), a molecule that mediates *M.tuberculosis* binding and internalization in human dendritic cells [23], was significantly downregulated in D21-infected lungs (Table 2). In contrast, we found that genes encoding C-type II lectins such as DECTIN-1 (CLEC7A), DECTIN-2 (CLEC4N) and Mincle (CLEC4E) were induced between D15 and D21 (Table 2), consistent with a role for DECTIN-1 in induction of inflammatory cytokines in macrophages following *M.tuberculosis* infection [24], [25]. Furthermore, Mincle was recently identified as the receptor for mycobacterial cord factor Trehalose dimycolate, which is involved in the induction of proinflammatory responses in myeloid cells [26]. Interestingly, the transcripts for C-type lectin MDL-1 (CLEC5A), which is mainly expressed by neutrophils and macrophages, were induced at D12 with considerable increase in their levels at D21 [27]. Furthermore, we found there was increased expression of the transcripts encoding CD11b (Integrin alpha M-ITGAM) and CD11c (Integrin alpha X-ITGAX) in D21 *M.tuberculosis*-infected lung, but not at earlier time points (Table 2).

We also found that mRNAs encoding the matrix metalloprotease, MMP13, was induced in D21-infected lungs (Table 2). Additional matrix metalloproteases, such as MMP9 and MMP8, were however not significantly induced at D21 in the *M.tuberculosis*-infected lungs (Table 2), although transcripts for MMP9 was induced early at D12. These data are consistent with the fact that MMPs are required for macrophage recruitment and granuloma formation [28] and suggest that these proteins may be involved in tissue remodeling and inflammation during early tuberculosis.

Following infection with *M.tuberculosis*, innate cells produce inflammatory cytokines that define the outcome of an adaptive host immune response. The induction of T helper 1 (Th1) responses during *M.tuberculosis* infection is largely dependent on IL-12 [12]. Accordingly, we found that mRNAs encoding IL-12p40 (IL12B) and its receptor IL-12RB1 were induced in D21 *M.tuberculosis*-infected lungs (Table 2). Furthermore, IL-6 plays a crucial role in driving T cell responses during tuberculosis [29], and we found a significant induction of IL-6 mRNA on day 21 post infection. In contrast, mRNAs encoding anti-inflammatory cytokines such as IL-4 and IL-10 were not induced in the *M.tuberculosis*-infected lung (Table 2). These data suggest that induction of mRNAs involved in the initiation of Th1 adaptive immunity takes place between D15 and D21 in the *M.tuberculosis*-infected lungs. Together, these data suggest that distinct molecular signatures of innate immunity are induced at different times during the early phases of *M.tuberculosis* infection and may function to activate downstream immune responses.

### Expression of mRNAs involved in adaptive Immune responses

**Figure 3:**
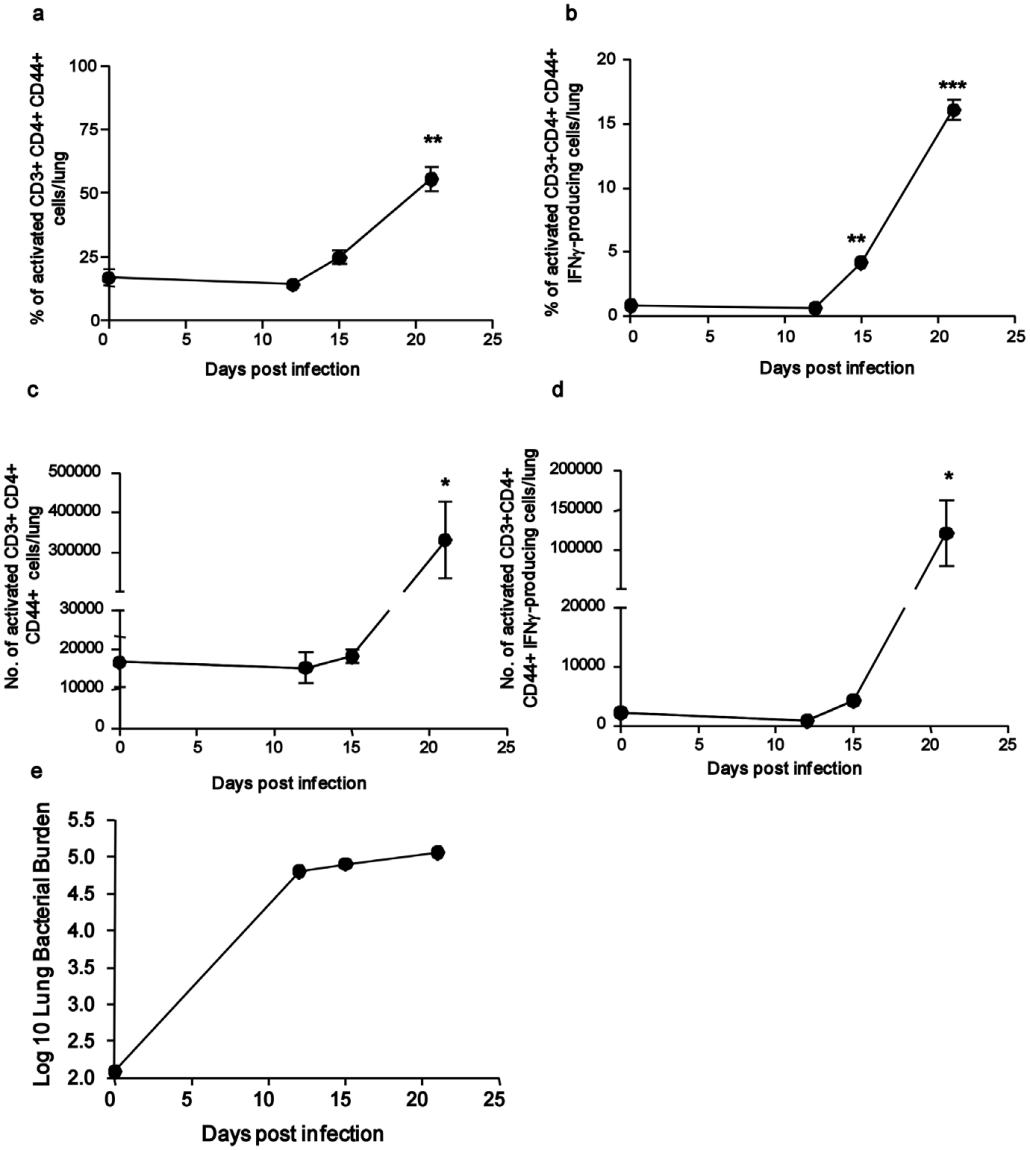
Accumulation of activated IFNγ-producing T cells in the lungs occurs between D15 and D21 following *M.tuberculosis* infection. B6 mice were infected with ∼100 CFU *M.tuberculosis* via the aerosol route and at specific times after infection lymphocytes were isolated from the lung to determine the percentage of activated CD4^+^ T cells (CD3^+^ CD4^+^ CD44^+^) (a), percentage of activated CD4^+^ T cell producing IFNγ (b) or number of activated CD4^+^ T cells (CD3^+^ CD4^+^ CD44^+^) (c), or number of activated CD4^+^ T cell producing IFNγ (d) by flow cytometry. The lung bacterial burden was determined in *M.tuberculosis*-infected lungs by plating lung homogenates (e). The data points represent the mean (±SD) of values from four-five mice (a–e). _*_, *p*≤0.05, _**_, *p*≤0.005. _***_, *p*≤0.0005 over Day 0 samples.

It is well known that the IFNγ-mediated activation of macrophages is critical for mycobacterial control [5]. Accordingly, we detected the induction of transcripts for IFNγ at D15 with further upregulation of IFNγ mRNA levels at D21 (Table 3). We next determined whether there was a positive correlation between induction of IFNγ mRNA in the lung between days 15 and 21, and the induction of genes linked to the IFNγ pathway. As expected, we found a positive correlation using Pearson's product moment correlation test, which follows a t-distribution with n-2 degrees of freedom, and found that genes encoding IFNγ-associated GTPases such as IGTP, IIGP1 and interferon-inducible proteins (IF130, IF135, IF144, IF147) as well as interferon regulatory factors (IRF1, IRF5, IRF7, IRF8) followed the pattern of IFNγ induction (Table 3). Furthermore, we found that transcripts for STAT-1, a key transcription factor in the IFNγ responses, was significantly induced at D21 (Table 3). To further validate the gene expression profiles, we then experimentally addressed the timing of accumulation of CD4^+^ T cells expressing IFNγ in *M.tuberculosis*-infected lungs by flow cytometry. We found that the percentage and number of activated CD4^+^ T cells (CD3^+^CD4^+^CD44^hi^) (Figure 3 a,c) and activated CD4^+^ T cells that produce IFNγ (Figure 3 b,d) accumulated in the *M.tuberculosis*-infected lungs between D15 and D21. These data suggest that Th1 cell accumulation occurs between D15–D21 in the mouse model of tuberculosis.

**Table 3:** Induction of genes associated with the IFNγ pathway during early *M.tuberculosis* infection.

EntrezID	Symbol	GeneName	D12		D15		D21		Corr.	p-val
			FC	q-val	FC	q-val	FC	q-val		
15978	IFNG	interferon gamma	1.02	1.26	**1.92**	**0.03**	**239.25**	**0.00**	-	**-**
76933	IFI27L2A	interferon, alpha-inducible protein 27 like 2A	1.19	0.47	**1.61**	**0.08**	**5.94**	**0.00**	0.96	**0.00**
65972	IFI30	interferon gamma inducible protein 30	−1.22	0.89	1.05	1.12	**2.59**	**0.02**	0.96	**0.00**
70110	IFI35	interferon-induced protein 35	−1.14	1.08	1.02	1.33	**2.01**	**0.05**	0.94	**0.00**
99899	IFI44	interferon-induced protein 44	−1.32	0.71	1.30	0.21	**11.48**	**0.00**	0.96	**0.00**
15953	IFI47	interferon gamma inducible protein 47	1.27	0.24	**1.73**	**0.03**	**7.79**	**0.00**	0.98	**0.00**
15957	IFIT1	interferon-induced protein with tetratricopeptide repeats 1	1.10	0.67	**1.75**	**0.04**	**15.27**	**0.00**	0.99	**0.00**
15958	IFIT2	interferon-induced protein with tetratricopeptide repeats 2	−1.24	0.81	1.26	0.25	**7.05**	**0.00**	0.99	**0.00**
15959	IFIT3	interferon-induced protein with tetratricopeptide repeats 3	−1.22	0.86	**1.53**	**0.10**	**8.52**	**0.00**	0.98	**0.00**
66141	IFITM3	interferon induced transmembrane protein 3	−1.09	1.29	−1.05	1.32	1.27	0.36	0.90	**0.00**
16145	IGTP	interferon gamma induced GTPase	1.19	0.23	**2.23**	**0.00**	**12.07**	**0.00**	0.96	**0.00**
60440	IIGP1	interferon inducible GTPase 1	−1.05	1.12	**2.40**	**0.00**	**17.86**	**0.00**	0.97	**0.00**
16362	IRF1	interferon regulatory factor 1	1.04	0.87	1.27	0.28	**3.58**	**0.01**	0.97	**0.00**
27056	IRF5	interferon regulatory factor 5	−1.10	1.14	1.18	0.60	**4.40**	**0.00**	0.97	**0.00**
54123	IRF7	interferon regulatory factor 7	−1.45	0.42	**1.55**	**0.09**	**23.29**	**0.00**	0.98	**0.00**
15900	IRF8	interferon regulatory factor 8	−1.48	0.42	1.04	0.78	**3.56**	**0.01**	0.97	**0.00**
20846	STAT1	signal transducer and activator of transcription 1	−1.16	0.99	1.44	0.12	**8.69**	**0.00**	0.97	**0.00**

Tumor Necrosis Factor-alpha (TNFα) is required for the formation of protective granulomas during tuberculosis [30]. We found that TNFα mRNA was expressed at D21 but not earlier in the *M.tuberculosis*-infected lungs (Table 4). Genes associated with the TNFα pathway, such as Tumor necrosis factor alpha-induced protein 2 (TNFAIP2), tumor necrosis factor receptor superfamily member 1b (TNFRSF1B), Fas ligand (FASL) and lymphotoxin (LTB) were all induced at D21 and showed a positive correlation with TNFα expression (Table 4). These data suggest that mRNAs associated with adaptive immune responses, such as those in the TNFα and IFNγ pathways are induced between D15–D21 following *M.tuberculosis* infection and coincide with control of *M.tuberculosis* burden in the lungs of infected mice (Figure 3e).

**Table 4:** Induction of genes associated with the TNFα pathway during early *M.tuberculosis* infection.

EntrezID	Symbol	GeneName	D12		D15		D21		Corr.	p-val
			FC	q-val	FC	q-val	FC	q-val		
21926	TNF	tumor necrosis factor	1.00	1.09	1.00	1.41	**16.68**	**0.00**	-	**-**
21928	TNFAIP2	tumor necrosis factor, alpha-induced protein 2	1.03	1.17	−1.08	1.26	**7.28**	**0.00**	**0.99**	**0.00**
21938	TNFRSF1B	tumor necrosis factor receptor superfamily, member 1b	−1.07	1.29	1.00	1.41	**3.95**	**0.01**	**0.99**	**0.00**
14103	FASL	Fas ligand (TNF superfamily, member 6)	−1.01	1.37	1.02	1.33	**23.22**	**0.00**	**0.97**	**0.00**
16994	LTB	lymphotoxin B	1.15	0.38	1.36	0.25	**4.32**	**0.00**	**0.92**	**0.00**
22029	TRAF1	TNF receptor-associated factor 1	**−3.66**	**0.00**	**−3.62**	**0.00**	**−2.88**	**0.09**	**0.74**	**0.00**
22031	TRAF3	TNF receptor-associated factor 3	−1.25	0.79	−1.21	0.78	1.58	0.15	**0.88**	**0.00**

Integrins CD11a and CD18 have been shown to be critical for recruitment of T cells to the lung and to confer host resistance following infection with aerosolized *M.tuberculosis* [31]. Accordingly, we found induction of transcripts encoding CD11a (ITGAL) and CD18 (ITGB2) on D21 in the *M.tuberculosis* infected lung (Table 5). Markers associated with T cells such as CD3 (CD3G, CD3D), CD8 (CD8A, CD8B1), CD4, and T cell activation molecules such as IL-7R and CD5 were also induced between D15 and D21 (Table 5). Interestingly, the induction of CD8 also coincided with the induction of granzymes (GZMA, GZMB, GZMK) (Table 5). Signaling lymphocyte activating molecule (SLAM) contributes to Th1 mediated immune responses to tuberculosis [32]. We found that SLAM genes, such as SLAM 6 and 7, were exclusively induced at D21 and that SLAM 8 was induced on both D15 and D21. The cell surface glycoprotein 2B4 (CD244) is related to CD2 and is implicated in the regulation of NK- and T-cell function [33] and it was expressed on D21, but not earlier during infection (Table 5). Furthermore, we also found that the invariant TCR zeta chain (CD247), a member of the CD3 complex which is associated with the clonotypic alpha/beta TCR hetrodimer is also induced in the *M.tuberculosis*-infected lungs at D15 and D21. It has been reported that Lymphocyte antigen 6 complex, locus I (LY6I), a maturation marker for T and B lymphocytes [34] is induced during murine tuberculosis [17], and we show that transcripts encoding LY6I is induced during the early immune response on D15, with progressively more induction of D21 (Table 5).

**Table 5:** Induction of genes associated with the adaptive immune responses during early *M.tuberculosis* infection.

EntrezID	Symbol	GeneName	D12		D15		D21	
			FC	q-val	FC	q-val	FC	q-val
16408	ITGAL	integrin alpha L	−1.24	0.85	−1.11	1.08	**3.21**	**0.01**
16414	ITGB2	integrin beta 2	−1.21	0.88	−1.12	1.06	**2.67**	**0.02**
12502	CD3G	CD3 antigen, gamma polypeptide	1.20	0.44	**1.57**	**0.07**	**27.92**	**0.00**
12500	CD3D	CD3 antigen, delta polypeptide	1.42	0.12	**2.59**	**0.00**	**22.00**	**0.00**
12504	CD4	CD4 antigen	1.00	1.44	1.00	1.09	**9.70**	**0.00**
12526	CD8B1	CD8 antigen, beta chain 1	−1.28	0.46	**1.75**	**0.07**	**18.83**	**0.00**
12525	CD8A	CD8 antigen, alpha chain	1.33	0.17	**2.11**	**0.01**	**64.63**	**0.00**
16197	IL7R	interleukin 7 receptor	−1.41	0.49	1.03	1.09	**2.58**	**0.02**
12507	CD5	CD5 antigen	**1.47**	**0.10**	**1.93**	**0.02**	**62.89**	**0.00**
14938	GZMA	granzyme A	1.03	0.40	1.47	0.15	**5.48**	**0.00**
14939	GZMB	granzyme B	−1.20	0.64	**1.63**	**0.08**	**31.58**	**0.00**
14945	GZMK	granzyme K	−1.07	1.16	1.49	0.38	**182.09**	**0.00**
74748	SLAMF8	SLAM family member 8	1.07	0.45	**3.09**	**0.00**	**132.33**	**0.00**
75345	SLAMF7	SLAM family member 7	1.04	1.27	1.27	0.23	**15.03**	**0.00**
30925	SLAMF6	SLAM family member 6	−1.34	0.62	1.04	1.19	**4.21**	**0.01**
18106	CD244	CD244 natural killer cell receptor 2B4	−1.17	0.72	1.04	0.69	**4.86**	**0.00**
12503	CD247	CD247 antigen	1.29	0.20	**1.54**	**0.07**	**7.87**	**0.00**
57248	LY6I	lymphocyte antigen 6 complex, locus I	1.33	0.14	**10.93**	**0.00**	**305.70**	**0.00**
246256	FCGR4	Fc receptor, IgG, low affinity IV	−1.05	0.93	1.42	0.23	**21.65**	**0.00**
14127	FCER1G	Fc receptor, IgE, high affinity I, gamma polypeptide	−1.25	0.86	1.06	1.04	**3.36**	**0.01**
14129	FCGR1	Fc receptor, IgG, high affinity I	**1.53**	**0.08**	**2.18**	**0.01**	**19.89**	**0.00**
14130	FCGR2B	Fc receptor, IgG, low affinity IIb	−1.03	1.22	1.08	0.94	**2.81**	**0.02**
14131	FCGR3	Fc receptor, IgG, low affinity III	1.06	0.88	1.18	0.58	**3.62**	**0.01**

Fc Gamma (FcG) receptors are important for the effector functions of antibodies and modulating immune responses [35]. In immune responses to *M.tuberculosis*, the absence of inhibitory receptor, FcGRIIb (encoding for FcG2B) during *M.tuberculosis* infection results in reduced bacterial burdens and decreased pathology, while absence of both inhibitory and stimulatory FcG receptors results in increased susceptibility and is associated with immunopathology [36]. We found that FcgRI (FCGR1) is induced in the lung at D12 and its expression was further upregulated at D15 and D21, while other FcG receptors such as FcGrIIB (FCGR2B), FcGrIII (FCGR3) and FcGrIV (FCGR4) are induced at D21 (Table 5). These data suggest that both inhibitory and stimulatory FcG receptors are induced in the lung during the early immune response following *M.tuberculosis* infection.

### Expression of mRNAs involved in host defense mechanisms

**Figure 4:**
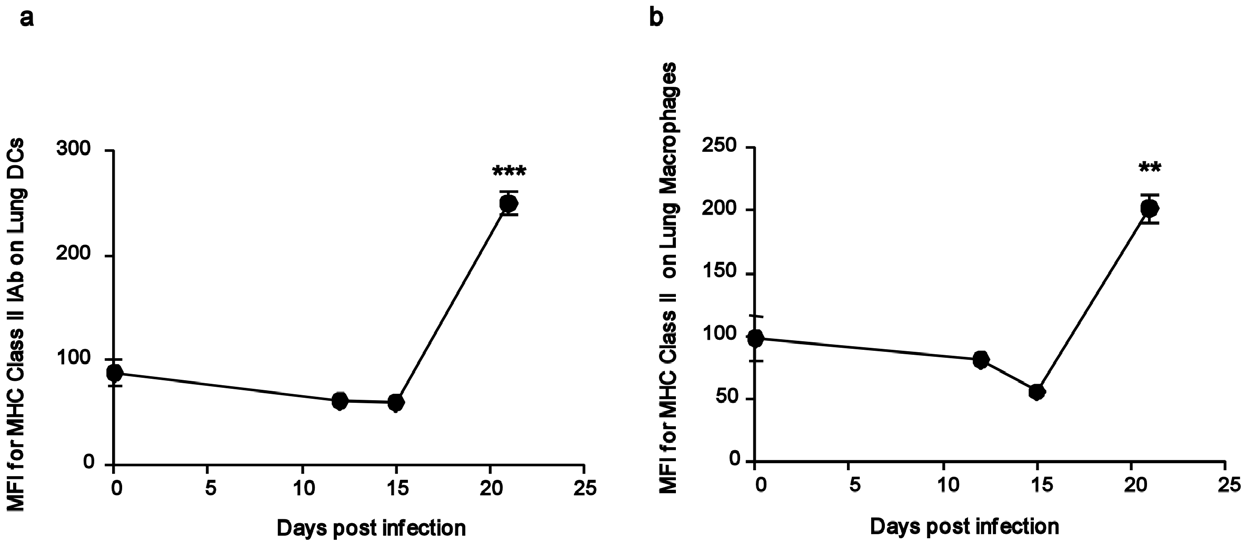
Upregulation of MHC Class II expression on lung myeloid cells occurs on D21 following *M.tuberculosis* infection. B6 mice were infected with ∼100 CFU *M.tuberculosis* via the aerosol route and at specific times after infection, cells were isolated from the lung to determine the expression of MHC Class II expression on lung DCs (a) or lung macrophages (b) by flow cytometry. The mean fluorescent intensity (MFI) of MHC Class II expression is shown. The data points represent the mean (±SD) of values from four-five mice (a, b). _**_, *p*≤0.005. _***_, *p*≤0.0005 over Day 0 samples.

Our data suggest that in the low dose mouse tuberculosis infection model, IFNγ mRNA is induced between D15 and D21 (Table 3), which coincides with the accumulation of activated IFNγ-producing T cells (Figure 3). IFNγ-mediated activation of macrophages and production of effector molecules, such as inducible nitric oxide synthase (NOS2) and the phagocyte oxidase (PHOX), are the major sources of reactive intermediates that are required for macrophage killing of *M.tuberculosis* [3], [37]. Interestingly, we found that transcripts encoding phagocyte oxidases (NCF1, NCF-2 and NCF4) were much more highly induced at D21 compared to NOS-2 mRNA transcripts (Table 6). In addition, mRNAs belonging to the MHC Class II pathway, such as MHC Class II transactivator (CIITA), which is the key molecule for MHC Class II expression [38], were also induced at D21 post infection (Table 6). Cellular analysis of *M.tuberculosis*-infected lung suspensions confirmed that upregulation of MHC Class II expression on lung DCs and lung macrophages occurs on D21 (Figure 4a,b). Mice lacking LRG-47 fail to control *M.tuberculosis* infection due to an iNOS-independent defect in maturation of *M.tuberculosis*-containing phagosomes [39] and we found that induction of the (p47) guanosine triphosphatase family, LRG-47 (IRGM1) occurs between D15 and D21 in the *M.tuberculosis*-infected lungs (Table 6), suggesting that control of mycobacteria is taking place during this specific stage of infection. Furthermore, induction of LRG-47 is IFNγ-dependent [39] and is consistent with the arrival of activated cytokine producing cells in the infected lung (Figure 3) and timing of upregulation of MHC Class II surface expression on myeloid cells (Figure 4) on D21.

**Table 6:** Induction of genes associated with host defense mechanisms during early *M.tuberculosis* infection.

EntrezID	Symbol	GeneName	D-12		D-15		D-21	
			FC	q-val	FC	q-val	FC	q-val
18126	NOS2	nitric oxide synthase 2, inducible	1.00	1.09	1.00	1.09	1.21	0.62
17969	NCF1	neutrophil cytosolic factor 1	−1.27	0.74	−1.11	1.05	**3.33**	**0.01**
17970	NCF2	neutrophil cytosolic factor 2	−1.27	0.81	1.00	1.22	**2.23**	**0.04**
17972	NCF4	neutrophil cytosolic factor 4	1.09	0.67	1.23	0.50	**6.20**	**0.00**
12265	CIITA	class II transactivator	1.06	0.38	1.36	0.18	**7.06**	**0.00**
15944	IRGM1	immunity-related GTPase family M member 1	−1.05	1.27	**1.72**	**0.03**	**8.62**	**0.00**
11846	ARG1	arginase, liver	−1.03	1.32	1.04	1.24	**2.18**	**0.04**
11847	ARG2	arginase type II	1.26	0.32	1.52	0.11	**3.81**	**0.01**
17002	LTF	lactotransferrin	−1.06	0.76	−1.22	0.48	**4.25**	**0.00**
15930	IDO1	indoleamine 2,3-dioxygenase 1	1.21	0.37	**3.70**	**0.00**	**270.79**	**0.00**
16792	LAPTM5	lysosomal-associated protein transmembrane 5	−1.19	0.86	−1.07	1.19	**2.34**	**0.03**
12514	CD68	CD68 antigen	−1.29	0.74	−1.02	1.29	**2.51**	**0.02**
12508	CD53	CD53 antigen	1.23	0.33	1.06	0.76	**7.09**	**0.00**
67844	RAB32	RAB32, member RAS oncogene family	1.26	0.43	1.18	0.62	**2.93**	**0.01**
60533	CD274	CD274 antigen	−1.09	1.14	**1.92**	**0.01**	**18.60**	**0.00**
58205	PDCD1LG2	programmed cell death 1 ligand 2	−1.02	1.34	1.01	1.36	**15.14**	**0.00**
18566	PDCD1	programmed cell death 1	−1.01	1.37	−1.00	1.39	**4.98**	**0.00**

Recently, arginase has been shown to be induced during *M.tuberculosis* infection and provides an advantage to the pathogen by suppression of nitric oxide [40]. Accordingly, we found ARG1 and ARG2 mRNAs were induced in D21-infected lungs (Table 6). Furthermore, to survive in the hostile host environment generated in the granulomas, *M.tuberculosis* can acquire iron through lactotransferrin [41]. Consistent with this survival strategy of *M.tuberculosis*, lactoferrin (LTF) was induced in *M.tuberculosis*-infected lungs at D21.

A growing body of evidence supports the hypothesis that specialized subsets of dendritic cells expressing indoleamine 2,3 dioxygenase (IDO1-INDO), which catalyzes oxidative catabolism of tryptophan have profound effects on T cell proliferation, differentiation, effector functions, and viability [42]. Further, it was found that TB patients expressed high levels of INDO, which following anti-TB treatment, declined to levels detected in controls [43]. We found that INDO mRNA was induced early at D15 and to higher levels at D21 (Table 6). In addition, genes favoring Mycobacterial survival such as LAPTM5 (lysosomal-associated protein transmembrane), CD68 (macrosial lysosoamyl glycoprotein) and CD53 (membrane late endosomes) and RAB32 protein [44], [45] were induced at D21-*M.tuberculosis*-infected lungs (Table 6). Programmed death 1 (PD-1), an activation-induced inhibitory receptor is expressed on lymphocytes and monocytes and binds to their ligands, PD-L1 and PD-L2 downregulating T cell responses [46], specifically they can impact T cell proliferation and cytokine production during *M.tuberculosis* infection [47]. Strikingly, PD-L1 (CD274) and PD-L2 (PGCD1LG2) were both induced on D21 in the *M.tuberculosis*-infected lungs (Table 6). This suggests that *M.tuberculosis* can actively promote down-modulatory mediators to counteract Th1-type and innate immunity as an immunopathological strategy.

### Gr1^+^ cells accumulate prior to accumulation of IFNγ-producing cells during murine tuberculosis

Neutrophils are one of the major effector cells of innate immunity since they can recognize, phagocytose and kill microorganisms using effector mechanisms that involve secretion of reactive oxygen species and cytotoxic components. One of the most striking patterns of gene expression that was induced early in D12-*M.tuberculosis*-infected lungs was associated with genes involved in neutrophil recruitment and genes expressed by neutrophils. For example, genes that encode for CXC-chemokines such as CXCL1 (Gro-α), CXCL5 (Ena-78) and CXCL9 (MIG) were significantly induced at D12 and were induced to higher levels on D15 and D21 post infection (Table 7). CXCL1 is expressed in neutrophils following stimulation with *M.tuberculosis* [48]. Furthermore, both CXCL1 and CXCL5 can also act as potent chemoattractants for neutrophils [49]. Interestingly, both CXCL1 and CXCL5 are induced in response to IL-1β stimulation [49], [50] and IL-1β can also induce neutrophil attraction [51], [52]. Consistent with this role for IL-1β, we found significant induction of IL-1β in D12-*M.tuberculosis*-infected lungs. Expression of IL-1β is critical for host immunity to *M.tuberculosis*, since mice that lack IL-1β are highly susceptible to infection and succumb in the first 40 days following infection [53]. These findings suggest that induction of IL-1β and chemokines CXCL1, CXCL5 and CXCL9 take place early during murine tuberculosis infection and may promote early recruitment of neutrophils to the *M.tuberculosis*-infected lungs.

**Table 7:** Induction of genes associated with early recruitment of neutrophil and monocyte during *M.tuberculosis* infection.

EntrezID	Symbol	GeneName	D-12		D-15		D-21	
			FC	q-val	FC	q-val	FC	q-val
14825	CXCL1	chemokine (C-X-C motif) ligand 1	**1.70**	**0.04**	**4.58**	**0.00**	**118.27**	**0.00**
20311	CXCL5	chemokine (C-X-C motif) ligand 5	**19.07**	**0.00**	**9.47**	**0.00**	**192.71**	**0.00**
17329	CXCL9	chemokine (C-X-C motif) ligand 9	**2.78**	**0.00**	**93.97**	**0.00**	**2134.40**	**0.00**
15945	CXCL10	chemokine (C-X-C motif) ligand 10	1.39	0.13	**20.54**	**0.00**	**357.38**	**0.00**
16176	IL1B	interleukin 1 beta	**1.86**	**0.02**	**1.96**	**0.01**	**28.16**	**0.00**
20201	S100A8	S100 calcium binding protein A8 (calgranulin A)	**2.91**	**0.00**	**1.55**	**0.07**	**3.44**	**0.01**
20202	S100A9	S100 calcium binding protein A9 (calgranulin B)	**3.14**	**0.00**	**1.52**	**0.09**	**3.93**	**0.01**
20210	SAA3	serum amyloid A 3	**2.72**	**0.00**	**34.77**	**0.00**	**274.66**	**0.00**
20296	CCL2	chemokine (C-C motif) ligand 2	**1.51**	**0.07**	**4.45**	**0.00**	**232.36**	**0.00**
20304	CCL5	chemokine (C-C motif) ligand 5	−1.30	0.63	1.24	0.45	**8.95**	**0.00**
20306	CCL7	chemokine (C-C motif) ligand 7	1.12	0.59	**2.58**	**0.01**	**108.37**	**0.00**
20307	CCL8	chemokine (C-C motif) ligand 8	**1.82**	**0.02**	**3.97**	**0.00**	**61.61**	**0.00**
20293	CCL12	chemokine (C-C motif) ligand 12	**2.81**	**0.00**	**3.13**	**0.00**	**69.50**	**0.00**
20299	CCL22	chemokine (C-C motif) ligand 22	**1.44**	**0.10**	1.31	0.35	**3.48**	**0.01**

Serum amyloid proteins are induced during tissue injury, infection and inflammation and are detected during murine tuberculosis [54] and in human tuberculosis patients [55]. Furthermore, addition of purified amyloid proteins to alveolar macrophages can enhance killing of *M.tuberculosis in vitro* [54]. Strikingly, we found significant induction of transcripts encoding Serum amyloid protein 3 (SAA3) in the D12 *M.tuberculosis*-infected lungs (Table 7). Furthermore, IL-1β can induce SAA3 induction [56] and serum amyloid proteins which can in turn mediate priming of neutrophils via induction of reactive oxygen species [57], [58]. S100A8 and S100A9 are small calcium-binding proteins that are expressed in tuberculosis patients [59] and produced by activated neutrophils and monocytes. Our data show that both S100A8 and S100A9 are induced in the D12-*M.tuberculosis*-infected lungs (Table 7). Importantly, S100A8, S100A9, and S100A8/A9 have each been shown to induce neutrophil chemotaxis [60], [61]. Furthermore, IL-1β is known to be a potent inducer of S100 proteins [62]. These data suggest that induction of genes encoding serum amyloid proteins and S100 proteins on D12 correlates with expression of other neutrophil-associated genes early in *M.tuberculosis*-infected lungs.

In addition to early induction of CXC chemokines, we also show that induction of genes encoding C-C chemokines namely CCL-2 (MCP-1), CCL8 (MCP-2), CCL12 (MCP-5) and CCL22 (MDC) occurs on D12 and progressively increases between D15 and D21 post infection (Table 7). The C-C-chemokines are small molecular weight chemotactic proteins that play an important role in neutrophil recruitment during inflammatory conditions [63] and in macrophage/monocyte recruitment during *M.tuberculosis* infection [64]. These data suggest that early induction of genes associated with monocyte and neutrophilic recruitment takes place on D12 in the *M.tuberculosis*-infected lungs.

**Figure 5:**
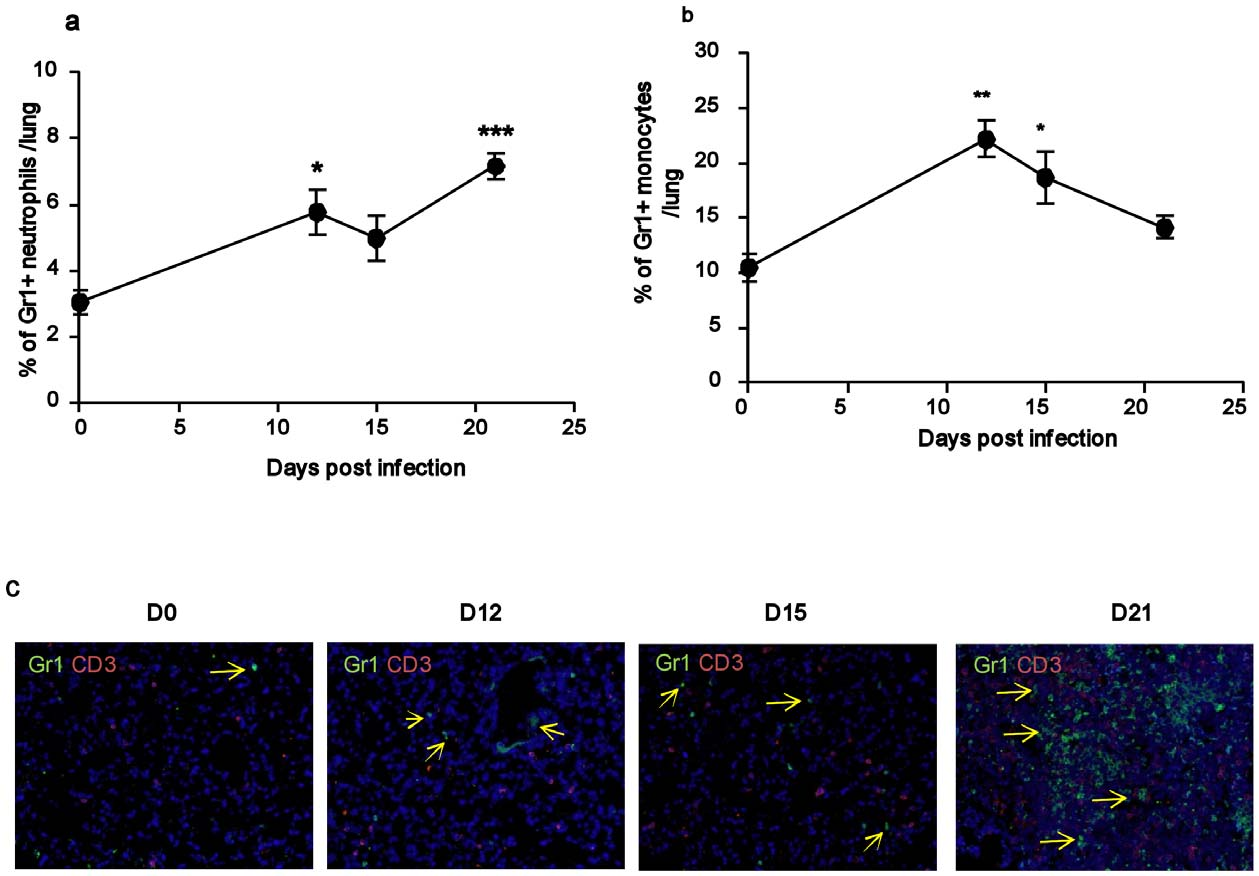
Early recruitment of Gr1^+^ cells occurs on D12 and precedes accumulation of activated T cells. B6 mice were infected with ∼100 CFU *M.tuberculosis* via the aerosol route and at specific times after infection, cells were isolated from the lung and the percentage of Gr1^+^ neutrophils (a) or Gr1^+^ monocytes (b) were determined by flow cytometry. In some B6 *M.tuberculosis*-infected mice, lungs were fixed in 10% formalin, embedded in paraffin and the accumulation of Gr1^+^ cells (green) and CD3^+^ T cells (red) determined by immunofluorescence (c). The data points represent the mean (±SD) of values from four-five mice (a,b). _*_, *p*≤0.05, _**_, *p*≤0.005. _***_, *p*≤0.0005 over Day 0 samples.

Our data suggest that genes associated with neutrophil and monocyte recruitment and activation are induced prior to induction of genes belonging to the adaptive immune response. Gr1 (Ly6G/Ly6C) is a marker that is expressed on both neutrophils as well as on a population of inflammatory monocytes [65], [66], [67]. For this reason, we experimentally determined the timing of accumulation of Gr1^+^ neutrophilic population (CD11b^+^ Gr1^+^) (Figure 5a) [68] and Gr1^+^ monocytic population (CD11b^−^ Gr1^+^ F4/80^+^) (Figure 5b) [65], [66], [67] in the *M.tuberculosis*-infected lungs. Consistent with our gene expression profiles, we found that Gr1^+^ neutrophilic and monocytic accumulation in the *M.tuberculosis*-infected lungs occurred at day 12 post infection (Figure 5a). While neutrophils were continuously recruited until D21, the accumulation of monocytes in the infected lungs was transient. Histologically, we observed an increased accumulation of Gr1^+^ cells around blood vessels on D12 and D15, while Gr1^+^ cells were observed in the lung interstitium by D21 (Figure 5c). These data experimentally support our gene expression analyses that early neutrophilic and monocytic responses precede the accumulation of activated T cells in the *M.tuberculosis*-infected lung.

### Depletion of Gr1^+^ cells during the early immune response results in reduced Th1 responses

**Figure 6:**
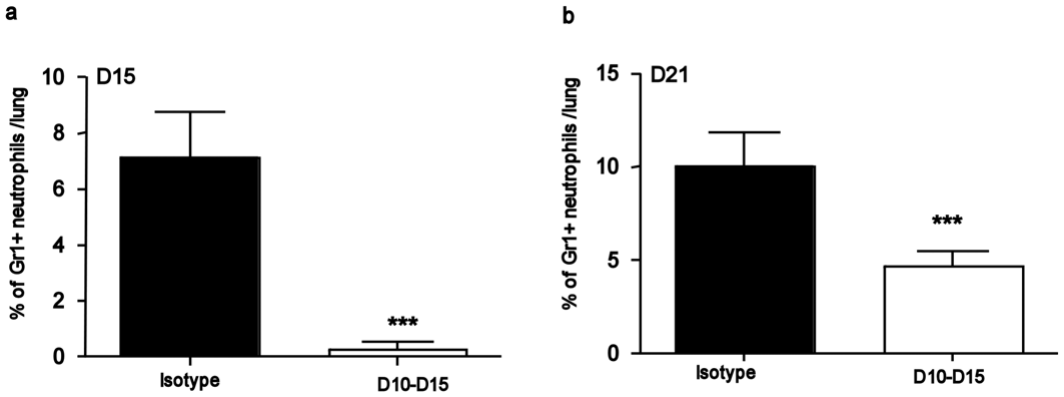
Treatment with Gr1+ depleting antibody results in significant decrease in lung neutrophils. B6 mice were infected with ∼100 CFU *M.tuberculosis* via the aerosol route and treated with either isotype control antibody (Isotype) or Gr1 depleting antibody between days 10–15 (D10–D15) post infection. Cells were isolated from antibody treated *M.tuberculosis*-infected mice and the percentage of lung neutrophils were determined on day 15 (a) or day 21(b) by flow cytometry. The data points represent the mean (±SD) of values from five mice (a–b) _***_, *p*≤0.0005 over isotype control treated *M.tuberculosis*-infected samples.

**Figure 7:**
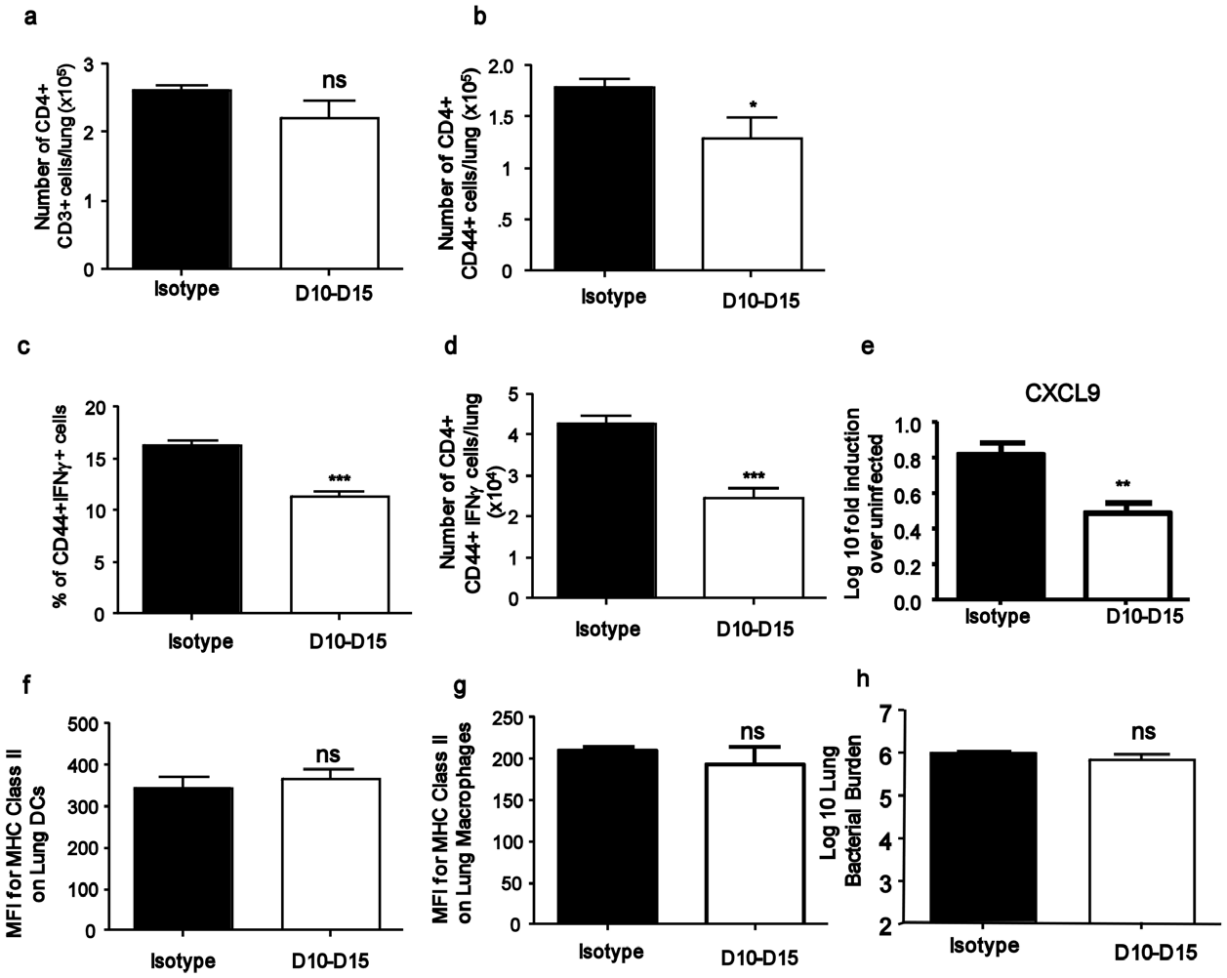
Depletion of Gr1^+^ cells between day 10–15 results in reduced Th1 responses but not impaired myeloid cell activation. B6 mice were infected with ∼100 CFU *M.tuberculosis* via the aerosol route and treated with either isotype control antibody (Isotype) or Gr1 depleting antibody between days 10–15 (D10–D15) post infection. Lung cells were isolated from antibody treated *M.tuberculosis*-infected mice and the number of total CD4^+^ CD3^+^ cells (a), number of activated CD4^+^ cells (b), percentage of activated IFNγ-producing cells (c), number of CD4^+^ CD44^+^ IFNγ-producing cells (d) was determined by flow cytometry. mRNA expression of CXCL9 in lungs of *M.tuberculosis*-infected isotype control mice or neutrophil-depleted mice was determined by RT-PCR (e). MHC Class II expression on lung DCs (f) and MHC Class II expression on lung macrophages (g) was determined in lungs of *M.tuberculosis*-infected isotype control treated mice or neutrophil-depleted mice by flow cytometry. The mean fluorescent intensity (MFI) of MHC Class II expression is shown. Lung bacterial burden from antibody-treated mice was determined by plating lung homogenates on D21 post infection (h). The data points represent the mean (±SD) of values from four-five mice (a–h). _*_, *p*≤0.05_**_, *p*≤0.005. _***_, *p*≤0.0005 over isotype control treated *M.tuberculosis*-infected samples. ns-not significant.

Given that our data suggesting that neutrophils and monocytes accumulate prior to activated T cells in the lung, we next addressed whether the early accumulation of Gr1^+^ neutrophils had an effect on host adaptive immune responses. To do this, we specifically depleted Gr1^+^ neutrophils using a monoclonal antibody (Clone 1A8), which is known to specifically deplete neutrophils without impacting Gr1^+^ monocyte populations [69]. We treated *M.tuberculosis*-infected mice with 1A8 antibody or isotype control antibody every 48 hours between D10–D15 post infection and this resulted in significant depletion of Gr1^+^ neutrophils in the lungs of Mtb-infected mice on D15 (Figure 6a). However, by D21 we could detect Gr1^+^ neutrophils, albeit at reduced frequency when compared to isotype-control treated mice (Figure 6b). Interestingly, depletion of Gr1^+^ neutrophils between D10–15 did not impact accumulation of total CD4^+^ T cells in the lungs (Figure 7a), but resulted in decreased accumulation of activated CD4^+^ cells (Figure 7b) and decreased percentage and number of IFNγ-producing T cells in the *M.tuberculosis*-infected lung on D21 (Figure 7c–d). Also, the reduced number of IFNγ-producing CD4^+^ T cells coincided with reduced expression of CXCL9 mRNA expression in the lung (Figure 7e). However, reduced number of IFNγ-producing cells did not have an adverse impact on activation of lung dendritic cells (Figure 7f), lung macrophages (Figure 7g) and lung bacterial burden (Figure 7h), since the MHC Class II upregulation and bacterial burden was comparable in control isotype-treated and Gr1-depleted *M.tuberculosis*-infected mice. Further, we did not find any differences histologically in granuloma formation (data not shown). These data suggest that the early recruitment of Gr1^+^ cells during *M.tuberculosis* infection plays a role in regulating the quality of adaptive CD4^+^ Th1 immune responses by modulating chemokine expression but does not impact overall protective outcomes.

## Discussion

A previous study analyzed the host immune response to *M.tuberculosis* infection and the progression to chronic TB infection using whole genome microarrays [17]. However, not much is known about the kinetics of the early host immune responses induced in the lung following *M.tuberculosis* infection. Using a low dose model of *M.tuberculosis* murine infection, we have comprehensively studied the early immune responses in the *M.tuberculosis*-infected lung. Our data show that mRNAs involved with heat shock proteins are amongst one of the first transcripts induced in the lung in response to *M.tuberculosis* infection. Interestingly, the host heat shock protein mRNA induction is transient and it is not maintained on D15. Furthermore, we also show that majority of the mRNAs encoding proteins that are involved in initiation of innate immune responses, such as Toll-like receptors and C-type lectins, were induced between D15 and D21. This pattern correlated with induction of mRNAs associated with generation of adaptive immune responses, such as markers from cells from the adaptive immune system and genes associated with production of effector cytokines. The induction of these mRNAs coincides with cellular accumulation of activated T cells that can produce IFNγ and activation of lung myeloid cells in the *M.tuberculosis*-infected lung on D21. These findings are consistent with published studies, in which adoptively transferred *M.tuberculosis*-specific T cells undergo activation and accumulate in the infected lungs between days 15–20 [8], [70]. Furthermore, we also show that accumulation of activated T cells in the lungs coincides with the induction of genes associated with host defense mechanisms such as PHOX and LRG-47, as well as genes associated with immunomodulatory functions such as arginase and INDO. These data suggest that there is a constant and dynamic regulation between host and pathogen factors that likely defines the final outcome of the infection.

One of the most intriguing questions in *M.tuberculosis* research is understanding what causes the delay in mounting an effective adaptive immune response, since accumulation of activated T cells does not occur until D15 in the *M.tuberculosis*-infected lungs. It has been proposed that it is likely that the slow growth of *M.tuberculosis* in the low dose aerosol challenge model results in limited availability of antigen, thereby resulting in a delay in antigen presentation and delayed accumulation of activated T cells in the lung [8], [70]. This is consistent with our data that show that genes involved with antigen presentation and pathogen associated receptor recognition are induced between D15 and D21, but not earlier. Another likely possibility for the delay in induction of mRNAs involved in antigen-presentation and initiation of T cell priming may be due to bacterial subversion of host immune responses. With regard to this, it has been shown that *M.tuberculosis* can inhibit MHC Class II presentation [71], [72] and it is possible that this results in delayed activation of antigen-presenting cells. Memory responses in previously infected mice [73] or in mice vaccinated with a *M.tuberculosis* vaccine [74], result in accumulation of activated T cells between D12 and D15 in the infected-lung. The small window of accelerated T cell memory responses results in enhanced control of bacterial burden and is reflected in 10 fold reduction in bacterial burden [73], [74]. These studies suggest that accelerating the accumulation of activated T cells to the lung may result in better protection outcomes. Studying the early memory responses using comprehensive gene and cellular profiling as described in this study will enable us to identify pathways induced early and will allow us to overcome the limitations of current vaccines by enhancing these innate mechanism and accelerating the initiation of adaptive immune responses.

One of the important and novel findings of this study is that genes associated with neutrophilic and monocytic responses are induced between D12 and D15 and precede the induction of genes associated with the Th1 pathway. Also, our experimental data showing the accumulation of Gr1^+^ neutrophils and monocytes on D12 in the *M.tuberculosis*-infected lung validates our gene expression profiles. To our knowledge, this is the first study to experimentally show that the accumulation of Gr1^+^ neutrophils in the lungs takes place prior to accumulation of adaptive T cells. The role of neutrophils in protective immunity against *M.tuberculosis* is controversial. Although accumulation of neutrophils in bronchoalveolar spaces has been described in active human tuberculosis [75], it is not known whether neutrophils have direct bacteriocidal or immunologic functions. Some *in vitro* studies have shown that neutrophils have direct mycobacteriocidal effects [76], [77], while other studies have shown that neutrophils fail to generate oxidative burst upon phagocytosis of mycobacteria [78]. In the mouse model of tuberculosis, depletion of neutrophils one day prior to infection did not impact overall susceptibility but had an effect on granuloma formation and resulted in delayed expression of CXCL9 [79]. The receptor for CXCL9, CXCR3 is expressed on activated T cells that are recruited to the *M.tuberculosis* infected lung [74]. Together, these data suggest that the early recruitment of Gr1+ neutrophils may play a role in regulating the adaptive T cell immunity during *M.tuberculosis* infection. This hypothesis is tested by depleting neutrophils between D10–15 and we show that this results in decreased expression of CXCL9 mRNA expression and reduced accumulation of Th1 cells in the *M.tuberculosis*-infected lung on D21. These data suggest that induction of neutrophil-associated chemokines such as CXCL9 and CXCL10 on D12–D15 may be key to accumulation of activated Th1 cells in the lung on D21. However, the decreased accumulation of Th1 cells on D21 did not impact overall myeloid cell activation or lung bacterial burden suggesting the Th1 response seen in neutrophil-depleted mice is likely sufficient to confer control of bacteria. Furthermore, we do not see any effects of neutrophil depletion in the pathology observed during the early phases in *M.tuberculosis*-infected lungs. These data suggest that the Th1 immune responses seen in isotype-treated *M.tuberculosis*-infected mice may in fact be excessive and may be contributing to the immunopathology associated with chronic TB during the later stages of infection. Based on these data, we are currently investigating whether the absence of early neutrophils has any effects on protective outcomes and pathology associated with chronic TB. This would shed further light on the detrimental (increased expression of CXCR3-ligating chemokines, Th1 recruitment and associated pathology) versus beneficial (optimal Th1 responses required for macrophage activation and mycobacterial control) effects of early neutrophil influx in TB pathogenesis.

Monocytes recruited to the site of infection can undergo differentiation into tissue macrophages or DCs. Recently a Gr1^+^ monocyte population was identified to be induced during inflammatory conditions and contributes to host resistance against pathogens [65], [66]. Consistent with this, we have identified that Gr1^+^ F4/80^+^ monocyte population accumulates in the lung early following *M.tuberculosis* infection. Unlike the Gr1^+^ neutrophil population, our studies show the Gr1^+^ monocyte population increase is transient, suggesting that these inflammatory monocytes probably then undergo differentiation into DCs and macrophages and contribute to effector function.

In summary, our cellular and gene expression analyses demonstrate the early recruitment of neutrophils and monocytes to the lung following *M.tuberculosis* infection. Further, we show that the early neutrophilic recruitment and associated gene expression likely contributes to the accumulation of activated cytokine-producing Th1 cells on D21. Further studies on which neutrophil-associated chemokines are crucial for regulation of adaptive immunity and will allow us to define immunopathology versus protection during TB.
